# Interface Modulation for the Heterointegration of Diamond on Si

**DOI:** 10.1002/advs.202309126

**Published:** 2024-03-13

**Authors:** Xing Li, Li Wan, Chaonan Lin, Wen‐Tao Huang, Jing Zhou, Jie Zhu, Xun Yang, Xigui Yang, Zhenfeng Zhang, Yandi Zhu, Xiaoyan Ren, Ziliang Jin, Lin Dong, Shaobo Cheng, Shunfang Li, Chongxin Shan

**Affiliations:** ^1^ Henan Key Laboratory of Diamond Optoelectronic Materials and Devices Key Laboratory of Material Physics School of Physics and Microelectronics Zhengzhou University Zhengzhou 450000 China; ^2^ School of Energy and Power Engineering Key Lab of Ocean Energy Utilization and Energy Conservation of Ministry of Education Dalian University of Technology Dalian 116024 China; ^3^ State Key Laboratory of Lunar and Planetary Sciences Macau University of Science and Technology Taipa Macao 999078 China

**Keywords:** β‐SiC, electron microscopy, heterointegration, interfacial structure, MPCVD diamond

## Abstract

Along with the increasing integration density and decreased feature size of current semiconductor technology, heterointegration of the Si‐based devices with diamond has acted as a promising strategy to relieve the existing heat dissipation problem. As one of the heterointegration methods, the microwave plasma chemical vapor deposition (MPCVD) method is utilized to synthesize large‐scale diamond films on a Si substrate, while distinct structures appear at the Si‐diamond interface. Investigation of the formation mechanisms and modulation strategies of the interface is crucial to optimize the heat dissipation behaviors. By taking advantage of electron microscopy, the formation of the epitaxial *β*‐SiC interlayer is found to be caused by the interaction between the anisotropically sputtered Si and the deposited amorphous carbon. Compared with the randomly oriented *β*‐SiC interlayer, larger diamond grain sizes can be obtained on the epitaxial *β*‐SiC interlayer under the same synthesis condition. Moreover, due to the competitive interfacial reactions, the epitaxial *β*‐SiC interlayer thickness can be reduced by increasing the CH_4_/H_2_ ratio (from 3% to 10%), while further increase in the ratio (to 20%) can lead to the broken of the epitaxial relationship. The above findings are expected to provide interfacial design strategies for multiple large‐scale diamond applications.

## Graphical abstract

The competitive growth between β‐SiC and non‐diamond carbon (C) phases at distinct CH_4_ concentrations is uncovered to occur prior to the diamond formation during the MPCVD process, resulting in various interfacial structures. The solid experimental observations evidence that the reaction between sputtered Si atoms from the Si substrate and the amorphous C nanorod leads to the epitaxy of β‐SiC nanoislands.

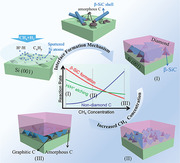

## 1 Introduction

Miniaturization of electronic devices and their heterointegration on Si substrates have become the main factors that determine the destiny of devices. Diamond, with many attractive properties, has been widely used in the fields of abrasive tools, high flux heat sinks, gas sensors deep ultraviolet detectors, etc.^[^ ^1^, ^2^, ^3^, ^4^, ^5^, ^6^, ^7^, ^8^ ^]^ Nowadays, the cost of diamond synthesis has decreased sharply, and diamonds become more acceptable for different usage scenarios. Integrating diamond with Si and/or Si‐based devices can not only accelerate the large‐scale applications of diamond, but also boost the functional properties of Si‐based devices. Especially, because of its high thermal conductivity, diamond is an excellent candidate to dissipate heat away from the hot spots in power electronics, and these power electronics are usually based on the well‐established complementary metal‐oxide semiconductor (CMOS) technology.^[^ ^9^, ^10^, ^11^, ^12^, ^13^, ^14^, ^15^ ^]^ The bottleneck for heat transfer in a diamond‐Si device is encountered at the interface.^[^ ^16^ ^]^ The quality and thickness of the interfacial layer between diamond and Si contribute significantly to the effective thermal boundary resistance.^[^ ^17^ ^]^ Replacing the amorphous interlayer with a higher thermal conductivity crystalline material like SiC (≈400 W m^−1^ K^−1^) would reduce the thermal boundary resistance and help to enable the full potential of Si‐based devices.^[^ ^18^, ^19^ ^]^ Moreover, for the diamond‐based gas/temperature sensor, optimization of diamond growth on Si substrate is essential for their fabrication and performance enhancement.^[^ ^20^ ^]^ In short, it is crucial for diamond to be compatible with the CMOS technique, and the interfacial structures between diamond and Si substrate need to be carefully studied.

Due to the low temperature, high rate, and high stability of the deposition process, the microwave plasma chemical vapor deposition technique (MPCVD) has been regarded as the most promising technique for homogeneously synthesizing large‐area, low‐cost, and high‐quality diamonds for device applications.^[^ ^21^, ^22^, ^23^, ^24^, ^25^, ^26^ ^]^ During the deposition process, the produced plasma can dissociate H_2_ into atomic hydrogen and activate hydrocarbon radicals (C_x_H_y_) to promote diamond formation through the reconstruction of carbon (C) atoms.^[^ ^27^, ^28^, ^29^, ^30^ ^]^ During the diamond deposition on Si substrate, the interfacial reactions are quite important for nucleation and device property optimization.^[^ ^31^, ^32^ ^]^ However, interlayers of amorphous carbon (a‐C), polycrystalline β‐SiC nanocrystallites, and epitaxial β‐SiC film have been observed to exist at the diamond–Si interface.^[^ ^33^, ^34^, ^35^, ^36^, ^37^, ^38^ ^]^ Due to the lack of investigation into their formation mechanisms, effective control strategies toward the Si‐diamond interface remain unsolved. The β‐SiC formation process has been speculated as a competitive C reaction channel during the MPCVD process^[^ ^39^, ^40^, ^41^ ^]^ and two distinct β‐SiC formation mechanisms have been proposed: I) the heteroepitaxial β‐SiC formation was caused by the direct C diffusion into the Si substrate; II) the embedded unoriented β‐SiC nanocrystallites are probably formed by the redeposition of etched Si.^[^ ^34^, ^39^, ^42^ ^]^ To date, there still lacks solid experimental evidence on the β‐SiC formation mechanism and its competitive reactions with other C phases. Therefore, experimentally revealing the C evolution is critical to elucidate the interfacial structure formation under MPCVD conditions.

Herein, we performed a series of short‐term growth of diamond films on Si substrate and revealed the interfacial reactions on Si substrate at the initial MPCVD process. Formation of epitaxial β‐SiC nanoislands with exposed {111} facets at the diamond–Si interface was observed at low CH_4_ concentrations. The epitaxial β‐SiC nanoislands were formed through the reaction between sputtered Si atoms and the a‐C nanorod. Our results suggest that the orientation and morphology of β‐SiC nanoislands can influence the subsequent diamond growth significantly. Moreover, we discovered that the distinct diamond‐Si interfacial structures were induced by the competitive growth between β‐SiC and non‐diamond C phases at different CH_4_ concentrations. The present findings can provide deep insight into understanding and controlling the atomistic interfacial reactions during diamond synthesis.

## 2 Results and Discussion

### 2.1 Epitaxial β‐SiC Nanoislands at the Diamond–Si Interface

**Figure 1:**
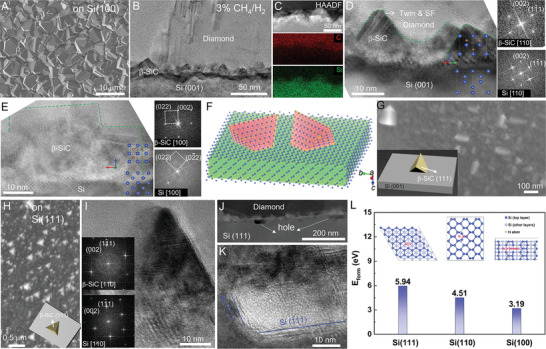
Morphology and orientation relationship between β‐SiC and Si substrate. A) SEM image of the synthesized β‐SiC nanocrystals on Si. B) Low‐mage TEM image and C) elemental mappings of the cross‐section of the diamond–SiC–Si interface after 5 h of MPCVD growth. HRTEM images of the SiC–Si interface viewed along D) Si [110] and E) Si [100] zone axes. F) Schematic illustration and G) High‐mag SEM image showing the morphology and orientation relationship between SiC nanocrystals and Si substrate. H) SEM, I) HRTEM image and corresponding Fast Fourier transformation (FFTs) viewed along the [110] zone axis of Si and β‐SiC nanoislands. J)High‐angle annular dark‐field (HAADF)‐STEM image of the diamond–β‐SiC–Si interface. K) HRTEM image of the hole structure in the Si (111) substrate. L) Calculated formation energy of a Si vacancy defect (E_d_) on the Si (111), (110) and (100) surfaces.

To understand the orientation relationship between the β‐SiC nanoislands with Si substrate and verify the reaction route, we conducted a detailed structural analysis on the diamond‐Si interfacial region. **Figure** **1**A presents the morphology of the synthesized polycrystalline diamond film on Si (001) substrate after 5 h growth at 3% CH_4_/H_2_. The cross‐sectional scanning electron microscope (SEM) image (Figure S1a, Supporting Information) of the diamond film after 4h's growth at the same condition showed that the thickness of the synthesized diamond film is ≈6.03 µm, corresponding to the deposition rate of 1.5 µm h^−1^. To reveal the interfacial structure, a cross‐sectional sample (Figure 1B) was fabricated from the synthesized diamond sample with a focused ion beam (FIB), and many triangular‐shaped islands were observed at the interfacial region. Further scanning transmission electron microscopy energy‐dispersive X‐ray (STEM–EDX) mappings revealed evenly distributed C and Si in these islands (Figure 1C). The high‐resolution transmission electron microscopy (HRTEM) images viewed along Si [110] and [100] zone axis (Figure 1D,E) showed that the triangular‐shaped nanoislands are β‐SiC with a “cube‐on‐cube” orientation relationship (the crystallographic axes of Si and β‐SiC are parallel) with the Si substrate. The lattice mismatch (Si:5.43 Å vs β‐SiC:4.35 Å) was released through the frequently observed stacking faults and misfit dislocations in the β‐SiC nanoislands (Figure 1D; Figure S1b,c, Supporting Information). It should be mentioned that the deposited diamond doesn't form a specific orientation relationship with the interfacial β‐SiC layer and no interfacial stress exists at the β‐SiC–diamond interface. Figure 1F exhibits a schematic illustration of the orientation relationship between β‐SiC and Si (Figure S2, Supporting Information), in close agreement with the observed morphology of β‐SiC nanoislands (Figure 1G). Specifically, the β‐SiC nanoislands present four exposed {111} facets and one (001) surface in contact with the Si (001) substrate.

To further confirm the orientation relationship between β‐SiC and Si substrate, Si (111) substrate was used to grow diamond film with the same MPCVD condition. As shown in Figure 1H, the formed nanoislands presented a tetrahedral morphology. The nanocrystals were identified as β‐SiC sharing a “cube on cube” orientation with the Si substrate (Figure 1I). Accordingly, the exposed surface of the tetrahedral‐shaped β‐SiC nanocrystals are also {111} planes (inset in Figure 1H). Importantly, holes with exposed Si {111} surfaces can be frequently observed in Si under the formed β‐SiC nanoislands (Figure 1J,K; Figure S3, Supporting Information). To decipher the underlying mechanism of such an anisotropic feature of the holes, we performed density functional theory (DFT) calculations on the formation energies of a Si vacancy defect (E_d_) on the Si (111), (110), and (100) surfaces. The calculated E_d_ of the Si (111), (110), and (100) surfaces are 5.94, 4.51, and 3.19 eV, respectively. This indicates that the appearance of such anisotropy of the holes is related to the relatively high bonding energy of the Si atoms in the closest packed (111) planes (Figure 1L). Correspondingly, as compared to the cases of Si (100) and (110) surfaces, the Si atoms in the (111) planes are relatively more difficult to be etched by the plasma (Figure S4, Supporting Information). Therefore, the formation of β‐SiC nanocrystals is very probably caused by the reactions between the sputtered Si atoms and C. It should be mentioned that although the nucleation rate of diamond on the polished Si (001) is quite low (Figure S5a, Supporting Information), the β‐SiC nanoislands still exist at the diamond–Si interface (Figure S5b, Supporting Information), but with a relatively larger size than those grown on the scratched Si substrate (Figure S5c, Supporting Information).

### 2.2 Formation Mechanism of Epitaxial β‐SiC Nanoislands

**Figure 2:**
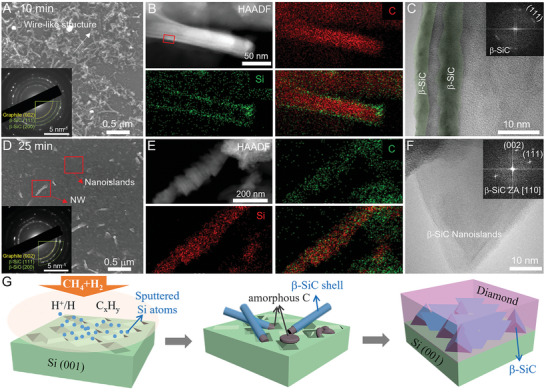
The formation mechanism of β‐SiC on the Si substrate during the initial MPCVD growth process. A) SEM image of the Si substrate after 10 mins’ growth; inset presents the SAED pattern of the formed nanostructures; B) HAADF‐STEM image and corresponding EDX mapping of C and Si and C) HRTEM images of the formed nanostructure in (A). D) SEM image of the Si substrate after 25 min of growth; inset presents the SAED pattern of the formed nanostructures; E) HAADF‐STEM image and corresponding EDX mapping of C and Si of the NW in D) and F) HRTEM images of the triangle‐shaped β‐SiC nanocrystals; G) Schematic illustration presenting the formation mechanisms of β‐SiC nanoislands during the initial MPCVD stage.

To reveal the formation mechanisms of the β‐SiC nanoislands, we performed a series of short‐term growth of diamond films on Si substrate and analyzed the microstructure evolution at the diamond–Si interface. **Figure** **2**A reveals the surface morphology of the Si substrate after 10 min MPCVD growth under 3% CH_4_/H_2_. The selected area electron diffraction (SAED) pattern evidenced the existence of β‐SiC and graphite nanocrystals in the wire‐like nanostructures. The STEM–EDX mappings of the wire‐like nanostructure present core‐shell distributed C and Si elements (Figure 2B). Further, HRTEM characterization (Figure 2C) showed that the wire‐like nanostructure consists of an a‐C core and a nanocrystalline β‐SiC (≈3–5 nm) shell. The graphitic C (g‐C) has also been detected during the HRTEM characterizations (Figure S6a, Supporting Information)).

After 25 min of growth, along with the decreased amount of the wire‐like nanostructures, many nanoislands formed on the Si substrate (Figure 2D; Figure S6b, Supporting Information). The existence of β‐SiC and graphite was also evidenced by the SAED pattern (inset in Figure 2D). As shown in Figure 2E, the STEM‐EDX mappings present the uniformly distributed Si and C in the formed nanowires (NWs). The TEM characterizations revealed the newly formed nanoislands possessed a triangular morphology with a size of ≈50 nm and were indexed to be β‐SiC (Figure 2F). Furthermore, the observed β‐SiC NWs almost vanished after 30 min of growth (Figure S7, Supporting Information). Regrowth of the wire‐like nanostructures can also be observed if we repeat the seeding growth process (Figure S8, Supporting Information). Therefore, it is concluded that, during the initial MPCVD growth stage, the C_x_H_y_ were reconstructed to g‐C and a‐C (mostly wire‐like structure). Our theoretical calculations predicated that Si atoms prefer to react with the activated a‐C (rather than g‐C) to form β‐SiC due to the higher binding energy of C_2_ (7.16 eV) than those of SiC (4.88 eV) and Si_2_ (3.61 eV) (Figure S9, Supporting Information). Then, the continuous reaction of sputtered Si atoms with a‐C to form β‐SiC nanocrystallites and finally form epitaxial β‐SiC islands with exposed {111} surfaces (Figure 2G).

### 2.3 Influence of β‐SiC on the Growth of Diamond Polycrystalline Film

**Figure 3:**
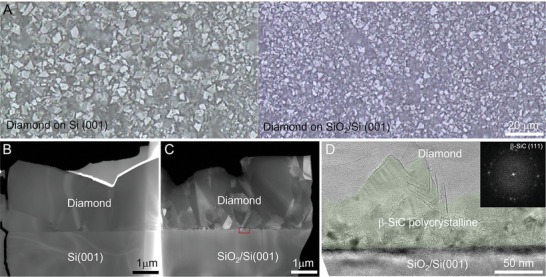
Influence of β‐SiC on the subsequent growth of diamond film. A) The optical image showing the morphology of diamond polycrystalline grown on Si (001) substrate with and without SiO_2_ pre‐deposition layer. Low‐mag HAADF‐STEM image of the cross‐sectional FIB sample fabricated from B) diamond on scratched Si (001) substrate and C) diamond on scratched Si (001) substrate with pre‐deposited SiO_2_ layer (Si (001)/SiO_2_). D) TEM image of the interfacial structure of diamond film grown on SiO_2_/Si (001).

To reveal the epitaxial relationship of β‐SiC and Si on the subsequent growth of polycrystalline diamond film, an amorphous SiO_2_ thin layer was pre‐deposited on half of the scratched Si (001) substrate (SiO_2_/Si(001)). After 5 h MPCVD growth, obvious differences in the grain size can be distinguished at the scratched and SiO_2_‐deposited regions (**Figure** **3**A). The HAADF‐STEM images of the cross‐sectional samples from both regions further confirmed that the diamond grown on SiO_2_/Si(001) presented a much smaller grain size than that grown on Si (001), especially at the interface region (Figure 3B,C). As presented in Figure 3D, due to the existence of deposited SiO_2_, the β‐SiC nanocrystals exhibited random orientations and sizes at the diamond‐SiO_2_/Si (001) interface (Figure S10a,b, Supporting Information). The diamond deposition rate on the SiO_2_/Si(001) is ≈1.7 times faster than that on the scratched Si(001) substrate (Figure S10c, Supporting Information). Therefore, our results suggest that the epitaxial relationship of β‐SiC and Si substrate can significantly influence the grain size of the synthesized diamond film. Due to the epitaxial relationship of β‐SiC with Si, changing the substrate orientations (from Si(001) to Si(111)) leads to distinct morphology of the β‐SiC: tetrahedron with three exposed {111} surfaces on Si (111) (Figure 1H) substrate and pentahedron with four exposed {111} surfaces on Si (001) substrate (Figure 1G). As compared in Figure S11 (Supporting Information), this morphology difference leads to a higher nucleation density and diamond film coverage on Si(001) than on Si(111). These observations suggest that the nucleation and quality of polycrystalline diamond film is closely related to the morphologies of β‐SiC between Si and diamond.

### 2.4 Competitive Growth of β‐SiC and Non‐Diamond C Phases at the Interfacial Region

A competing reaction between hydrogen etching of the non‐diamond phases and the formation of diamond simultaneously occurred depending on the concentration of CH_4_.^[^ ^43^, ^44^ ^]^ To uncover the influence of CH_4_ concentration on the interfacial reactions, we synthesized polycrystalline diamond films under CH_4_ contents of 3% (Figure 1), 10%, and 20% in CH_4_/H_2_ mixture while keeping other conditions the same. The morphology characterizations showed that, after the same growth period, the film thickness of the diamond increases with CH_4_ concentration (Figure S12a,d,g, Supporting Information). The diamond film grown on Si(111) substrate also presents the same trend (Figure S13, Supporting Information). Moreover, the X‐ray diffraction (XRD) spectra revealed that the diamond film synthesized at 20% CH_4_/H_2_ presented a distinct orientation with the film grown at 3% and 10% CH_4_/H_2_ (Figure S12c,f,i, Supporting Information).

**Figure 4:**
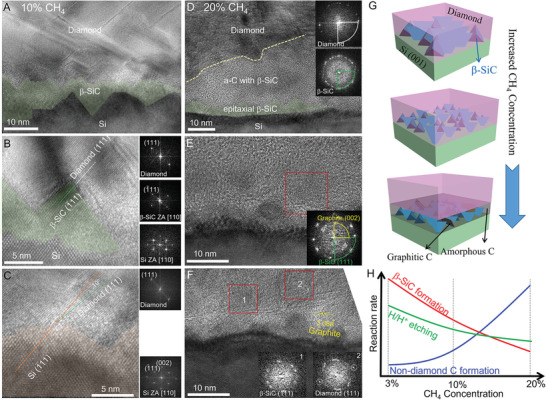
Interfacial structural analyses of the diamond films grown at A–C) 10% and D–F) 20% CH_4_ concentrations. G) Schematic illustration showing the interfacial structure synthesized at distinct CH_4_/H_2_ concentrations. H) The relationship between the reaction rates of H/H^+^ etching, β‐SiC, and non‐diamond C phase formation with CH_4_ concentrations.

To uncover the dominant factor leading to the distinct diamond film orientations, the detailed interfacial structures of diamond synthesized at 10% (**Figure** **4**A–C) and 20% (Figure 4D–F) CH_4_/H_2_ were analyzed as well. For diamond synthesized at 10% CH_4_/H_2_, the epitaxial β‐SiC nanoislands also exist at the interfacial region (Figure 4A), but exhibit a decreased size compared with that grown at 3% CH_4_/H_2_ (≈10 nm vs ≈20 nm, Figures 4A,B and 1D). Thus, we infer that the epitaxial growth of β‐SiC islands is slowed down at relatively high CH_4_ concentrations. Furthermore, the direct epitaxy of diamond on the Si without the β‐SiC formation was also discovered (Figure 4C). The diamond {111} prefers to align with the β‐SiC {111} and Si {111} with small misorientations (10°) (Figure 4B,C). When the CH_4_ concentration was further increased to 20%, in addition to a thin layer of epitaxial β‐SiC nanoislands (≈5 nm), a thick amorphous interlayer with randomly embedded β‐SiC nanocrystallites and g‐C layers were observed at the interface region (Figure 4D–F). STEM‐EDX mappings also present the distinct interfacial structure of diamond synthesized at 10% and 20% CH_4_/H_2_ concentrations (Figure S14, Supporting Information). The schematic illustrations of distinct diamond‐Si interfacial structures under various CH_4_ concentrations are presented in Figure 4G. It should be mentioned that due to the high reaction energy barrier in the formation of the Si─C bond from the Si atom and the C atom in g‐C (Figure S15, Supporting Information), the g‐C can be retained in the amorphous layer.

During the MPCVD process, H_2_ plasma is necessary to break C─H bonds and provide C_x_H_y_ for diamond growth. Moreover, H/H^+^ can etch non‐diamond phases during the diamond deposition process.^[^ ^45^, ^46^, ^47^ ^]^ It has been reported that the increased CH_4_ contents can lead to the increased formation of non‐diamond C phases. Along with the decreased etching ability of H/H^+^ on non‐diamond phases with the increased CH_4_ concentration, our results revealed a decreased size of β‐SiC at 10% CH_4_/H_2_ than that at 3% CH_4_/H_2_ (Figure 1B,D versus Figure 4A–C), suggesting that the formation rate of β‐SiC is decreased with CH_4_ concentration. At low CH_4_ concentrations (3% and 10%), the non‐diamond C phases can be etched effectively and timely by H/H^+^. Hence, only the Si‐SiC‐diamond and Si‐diamond interfacial structures were observed. At 20% CH_4_/H_2_, the formation rate of non‐diamond C phases is enhanced, while the etching ability of H/H^+^ is further decreased and becomes insufficient to etch the non‐diamond C phases. Hence, the epitaxial growth of β‐SiC is interrupted by the a‐C and g‐C layers, resulting in β‐SiC nanocrystals embedded in the amorphous layer.

The C_x_H_y_ can form g‐C and a‐C, while the a‐C can further form β‐SiC with sputtered Si atoms (Figure 2). When the etching ability of H/H^+^ is insufficient, the remaining a‐C can also transformed to g‐C under ≈880 °C.^[^ ^48^, ^49^ ^]^ Therefore, with increased CH_4_ concentrations, the decreased β‐SiC formation rate and H/H^+^ etching ability indicate an increased formation rate of non‐diamond C phases. Schematic illustrations showing the competitive growth of β‐SiC and non‐diamond C phases with CH_4_ concentrations are concluded in Figure 4H.

## 3 Conclusion

In summary, the evolution of C species prior to diamond formation during the MPCVD process has been revealed. The anisotropic sputtered Si atoms from the substrate by the plasma gradually react with the a‐C nanostructures and finally form epitaxial β‐SiC islands on the Si substrate. The “cube‐on‐cube” orientation relationship between β‐SiC and Si results in distinct β‐SiC morphologies on various Si substrates, which can affect the subsequent diamond growth severely. Importantly, the CH_4_ concentration was revealed to influence the interfacial structure through the competitive growth of β‐SiC and non‐diamond C phases. Our results show that the different growth conditions would largely affect the interfacial structure, and thus could influence the functional properties of the diamond films. These results can provide insight into the understanding of the interfacial reactions during the early MPCVD synthesis process and thus can benefit the interfacial design for the performance improvement of Si‐based diamond devices.

## 4 Experimental Section

### Synthesis of Diamond Film

The polycrystalline diamond films were grown on Si substrates using the MPCVD technique. Prior to the growth, the Si substrates were ultrasonically cleaned in acetone, methanol, and deionized water for 10 min successively and blow‐dried using high‐purity nitrogen. High‐purity (7 N) H_2_ and CH_4_ were employed as the reactant gases for the growth of diamond. During the growth process, the substrate temperature was set at ≈880 °C. The CH_4_ concentrations in the CH_4_/H_2_ mixture (228 mbar) were set to 3%, 10%, and 20%. To reveal the influence of the substrate on the diamond/Si interface, mechanical scratched Si(001) substrate, Si(111) substrate, polished Si(001) substrate, and mechanical scratched Si(001) substrate with deposited amorphous SiO_2_ were used, respectively.

### Characterizations

JEOL SEM was used to observe the morphologies of the samples at a low voltage (6 kV). The structures of the synthesized diamond films were evaluated by using XRD (X'Pert Powder). The cross‐sectional TEM samples were fabricated by an FEI Helios dual‐beam system. The samples were polished at low accelerating voltages to remove the surface damage layers at the last step of the sample preparation. The HRTEM images and EDX mappings were obtained on a JEOL 2100 TEM and a FEI Talos F200S at 200 kV, respectively.

### Theoretical Calculations

The spin‐polarized DFT calculations^[^ ^50^ ^]^ were performed using the Vienna ab initio simulation package^[^ ^51^, ^52^ ^]^ with the projector augmented wave method.^[^ ^53^, ^54^ ^]^ The generalized gradient approximation function of Perdew–Burke–Ernzerhof was employed for the exchange‐correlation energy.^[^ ^55^ ^]^ Based on comparison, the zero damping DFT‐D3 method of Grimme^[^ ^56^ ^]^ was adopted in describing van der Waals interactions. During the structural relaxation, all atoms were fully relaxed until the residual forces in each direction were ˂0.02 eV Å^−1^. The optimized lattice constants were 5.485 Å of the Si lattice, in close agreement with the experimental value of 5.43 Å.^[^ ^57^ ^]^ The electronic wave functions were expanded on a plane wave basis with an energy cutoff of 400 eV. Here, the Si substrates were simulated by a slab model with a vacuum region of 15 Å thickness to ensure decoupling between the neighboring images. The k‐space integration was performed with a 1 × 1 × 1 k‐point mesh in the Brillouin zone for the 5 × 5 × 1, 3 × 3 × 1 and 3 × 3 × 1 supercells of Si(111), Si(110) surfaces and Si(100)−2 × 1 reconstruction structures, respectively. Furthermore, the thermodynamic and kinetic properties of the etching process were investigated by hydrogen with the climbing‐image nudged elastic band (CI‐NEB) method.^[^ ^58^ ^]^

## Conflict of Interest

The authors declare no conflict of interest.

## Author Contributions

X.L., L.W., and C.L. contributed equally to this work. X.L., L.W., and C.L. conceived and designed the study. S.C., S.L., and C.S. supervised the project. C.L., X.Y., and Z.Z. performed the MPCVD synthesis of diamond films. X.L., L.W., S.C., and W.H. performed the characterization and analyses, including SEM, TEM, FIB, and XRD. Y.Z., X.R., and S.L. conducted theoretical calculations. J.Z., J.Z., Z.J., X.Y., and L.D. participated in discussions and provided suggestions for this work. All of the authors commented on the manuscript.

## Supporting information

Supporting Information

## Data Availability

The data that support the findings of this study are available in the supplementary material of this article.
