# Supplementary material for: Interface Modulation for the Heterointegration of Diamond on Si
Source: Adv Sci (Weinh). 2024 Mar 13;11(24):2309126. doi: 10.1002/advs.202309126 (PMC11199985; doi:10.1002/advs.202309126)
Supplement: Supplementary file 1 — Supporting Information [file ADVS-11-2309126-s001.pdf]

ADVANCED SCIENCE
Open Access

Supporting Information

for *Adv. Sci.*, DOI 10.1002/advs.202309126

Interface Modulation for the Heterointegration of Diamond on Si

Xing Li, Li Wan, Chaonan Lin, Wen-Tao Huang, Jing Zhou, Jie Zhu, Xun Yang, Xigui Yang, Zhenfeng Zhang, Yandi Zhu, Xiaoyan Ren, Ziliang Jin, Lin Dong, Shaobo Cheng, Shunfang Li* and Chongxin Shan**

Supporting Information

Interface Modulation for the Heterointegration of Diamond on Si

Xing Li[1,]†, Li Wan[1,]†, Chaonan Lin[1,]†, Wen-Tao Huang[1], Jing Zhou,[2] Jie Zhu,[2] Xun Yang[1], Xigui Yang[1], Zhenfeng Zhang[1], Yandi Zhu[1], Xiaoyan Ren[1], Ziliang Jin[2], Lin Dong[1], Shaobo Cheng[1,], Shunfang Li[1,]*, Chongxin Shan[1,]**

[1] Henan Key Laboratory of Diamond Optoelectronic Materials and Devices, Key Laboratory of Material Physics, School of Physics and Microelectronics, Zhengzhou University, Zhengzhou, 450000, China

[2] School of Energy and Power Engineering, Key Lab of Ocean Energy Utilization and Energy Conservation of Ministry of Education, Dalian University of Technology, Dalian 116024, China

[3] State Key Laboratory of Lunar and Planetary Sciences, Macau University of Science and Technology, Taipa, 999078, Macao, China

***Corresponding authors.** E-mails: chengshaobo@zzu.edu.cn; sflizzu@zzu.edu.cn; cxshan@zzu.edu.cn

† Equally contributed to this work.

Epitaxial relationship between β-SiC nanoislands and Si substrate. Figure S1 presents the deposition rate of the synthesized diamond film and the Si/β-SiC interfacial structure. Many misfit dislocations exist at the Si/β -SiC interface. Moreover, the stacking faults can also be frequently observed in the epitaxial β -SiC nanoislands.

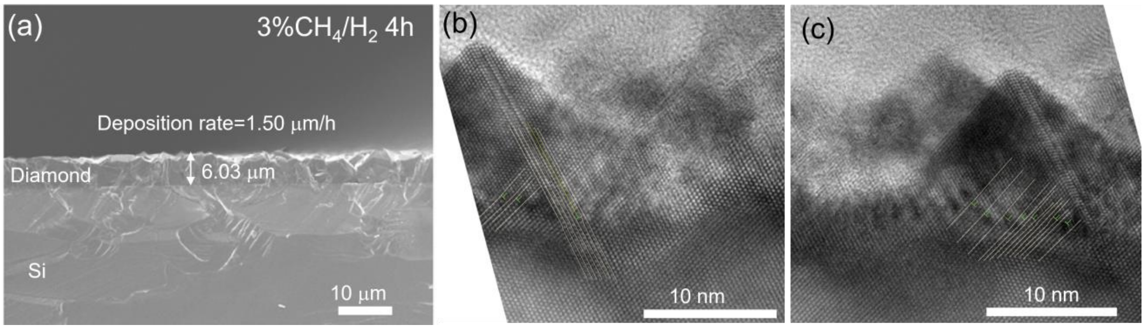

Figure S1. (a) The thickness and deposition rate of diamond on Si (001). (b,c)Stacking faults and misfit dislocations presented at the Si/ β-SiC interface.

Figure S2a presents the schematic illustration of the orientation relationship between β -SiC and Si substrate. Figure. S2b and c show the morphologies of the β-SiC nanoislands when viewed along the [110] and [100] zone axis, respectively. Figure S2 d,e and f are the corresponding HRTEM images of the β-SiC nanoisland when viewed along the [110] and [100] zone axis.

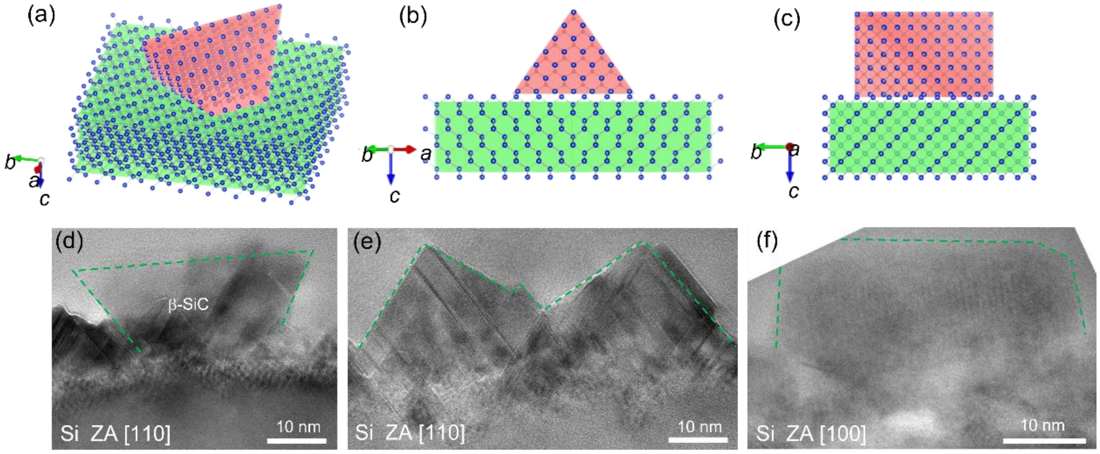

Figure S2. Schematic illustrations and HRTEM images presenting the morphology and orientation relationship of β-SiC and Si substrate. Green and red planes correspond to the Si (001) surfaces and β-SiC (111) surfaces.

Interfacial structure of β-SiC on Si(111) substrate. Figure S3a presents the cross

sectional STEM image of the diamond film grown the Si(111) substrate. As shown in Figure S3b, at the interface region, many holes exist in the Si substrate under the β-SiC nanocrystals. As indicated by the dashed yellow lines in Figure S3c, the holes are surrounded by the Si (111) surfaces.

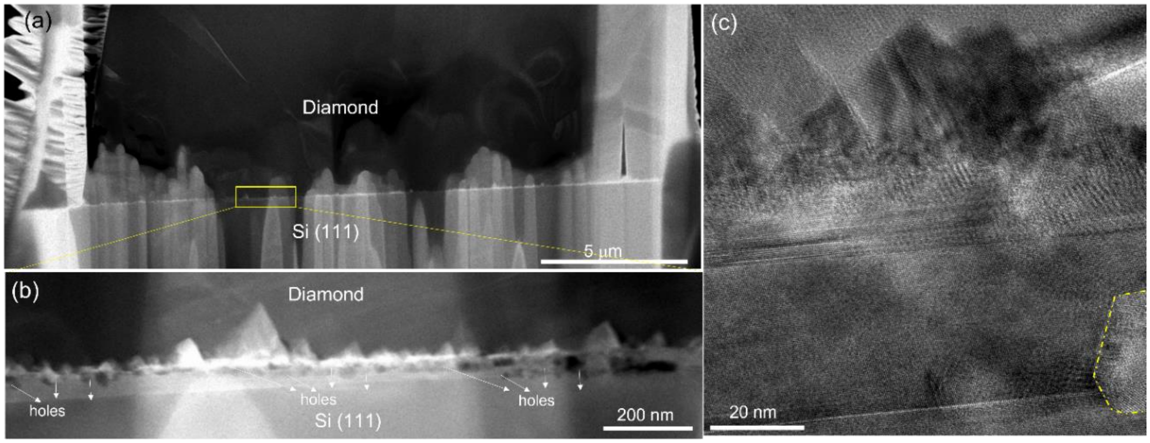

Figure S3. (a) The low-mag HAADF-STEM image of the cross sectional TEM sample fabricated from diamond grown on Si(111) substrate. (b) HAADF-STEM images showed the formed holds in the Si substrate under the β-SiC nanocrystals. (c) HRTEM image showed that the hole is formed by Si (111) surfaces.

Anisotropic etching of Si substrate by the H plasma. Figure S4 presents the theoretical calculations of the energy needed to remove a Si atom from the Si (111), (110) and (100) surfaces by a H atom. We can infer that Si (111) surface are relatively more difficult to be etched by the H plasma during the initial growth of diamond film during the MPCVD process.

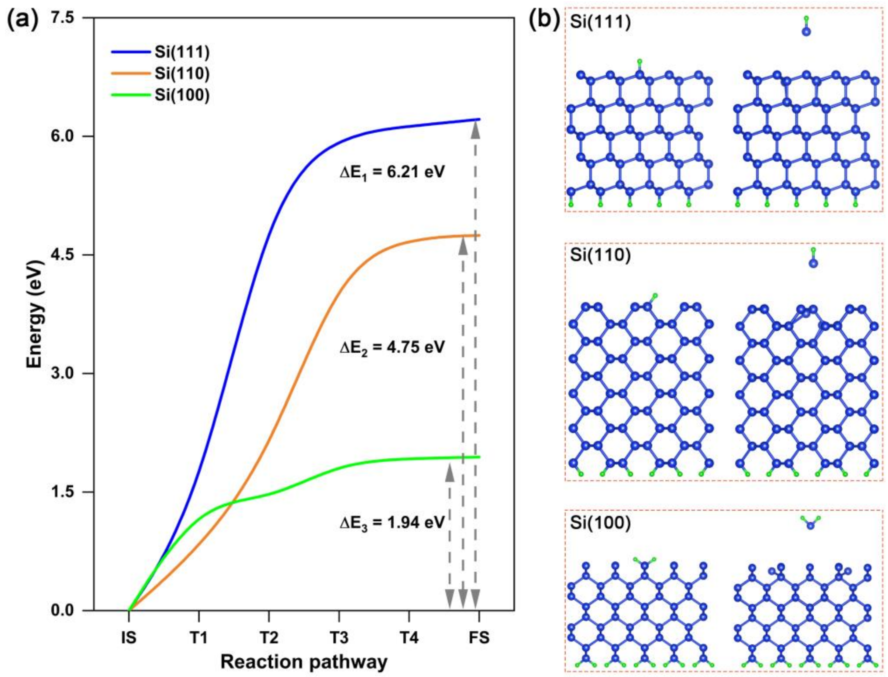

Figure S4. Theoretical calculations presenting the energy needed to remove a Si atom from the (111), (110) and (100) surfaces by H atom.

Interfacial structure of diamond grown on the polished Si (001) substrate. Figure S5a presents the morphology of the synthesized polycrystalline diamond film on the polished Si (001) substrate after 5 hours' growth at 3% CH_4/H_2. The nucleation rate of the diamond crystals is lower than that grown on the mechanical scratched Si (001) substrate (Figure 1A). However, the epitaxial β-SiC nanoislands present a relatively larger size (Figure S5b,c).

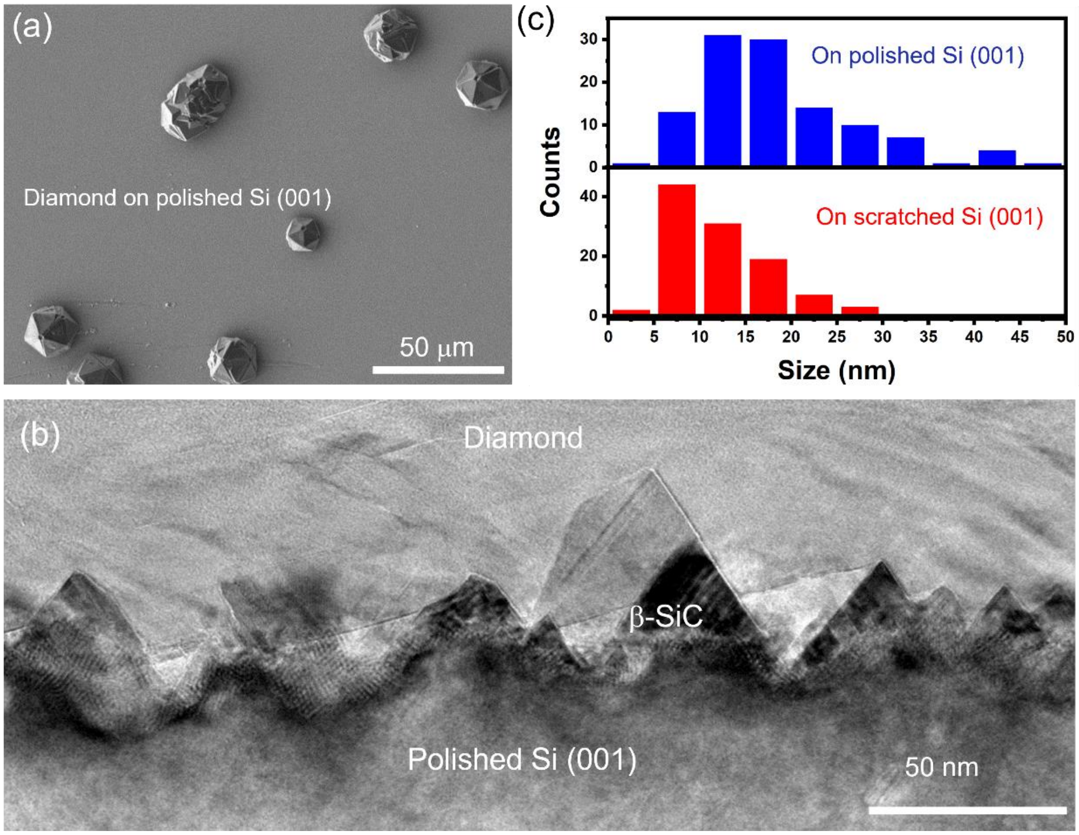

Figure S5. Formation of β-SiC islands on the polished Si (001) substrate. (a) SEM image of the synthesized diamond nanocrstals. (b) HRTEM image of the interfacial structure. (c) Size distribution of the β-SiC island on the polished and mechanical scractched Si (001) substrate.

Formation mechanism of epitaxial β-SiC nanocrystals. Figure 6a presented the HRTEM image of the synthesized materials after 10 min's growth, from which graphitic carbon can be clearly observed. Figure S6b is the STEM image presenting the morphology of the nanoislands on the Si surface after 25min's growth.

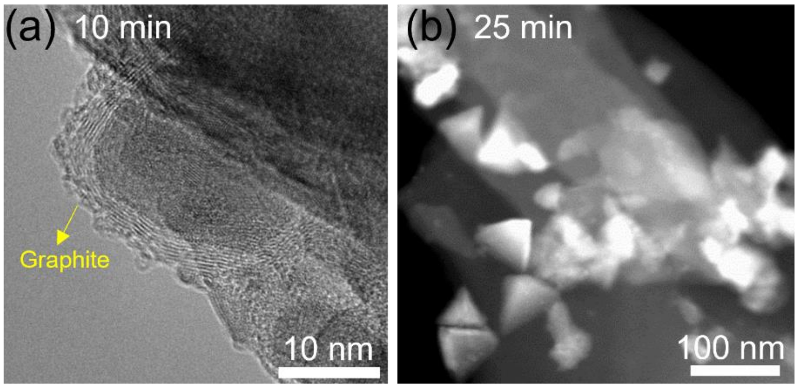

Figure S6. (a) HRTEM image of the formed nanostructures after 10mins' growth. (b) HAADF-STEM image of the triangular-shaped β-SiC nanocrystals after 25 mins' growth.

Figure S7 presents the morphology of the synthesized materials on Si surface after (a) 10 mins', (b) 25 mins' and (c) 30 mins' MPCVD growth. Specifically, the formed nanowires vanished after 30 min's growth (Figure. S7b *vs.* c).

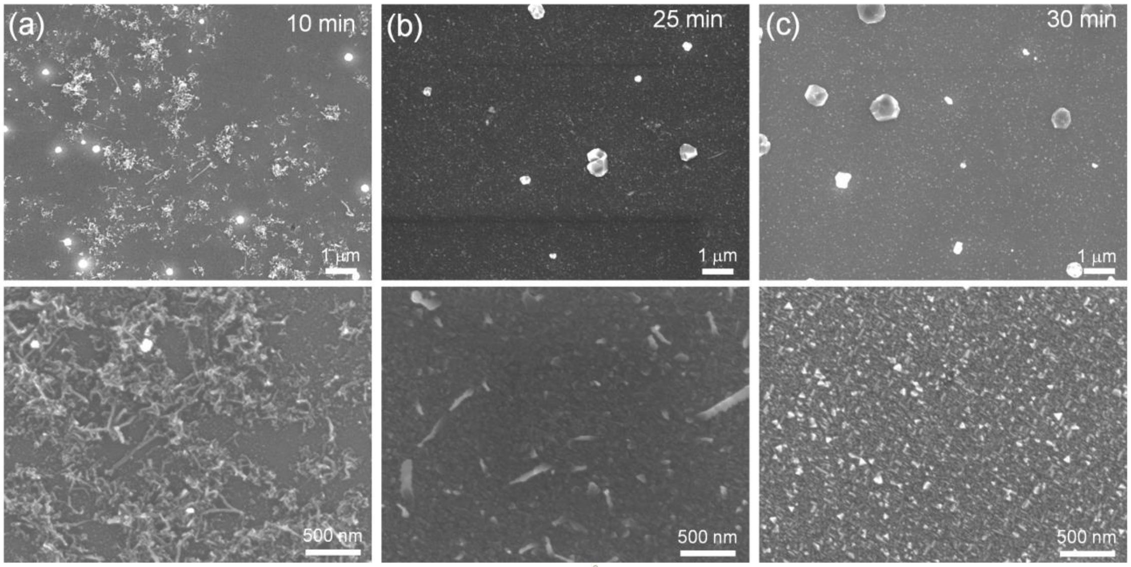

Figure S7. Morphology of the synthesized materials on Si substrate after (a)10 mins', (b) 25 mins' and (c) 30 mins' growth with MPCVD method.

SEM images of the synthesized materials on the Si (001) substrate after 5 growth process. The growth time was set to 5 mins at each growth process, and the substrate

temperature was set at around 880 ºC. The CH_4 concentrations in the CH_4/H_2 mixture (228 mbar) was set to 3%. The regrowth of the wire-like nanostructures can be observed.

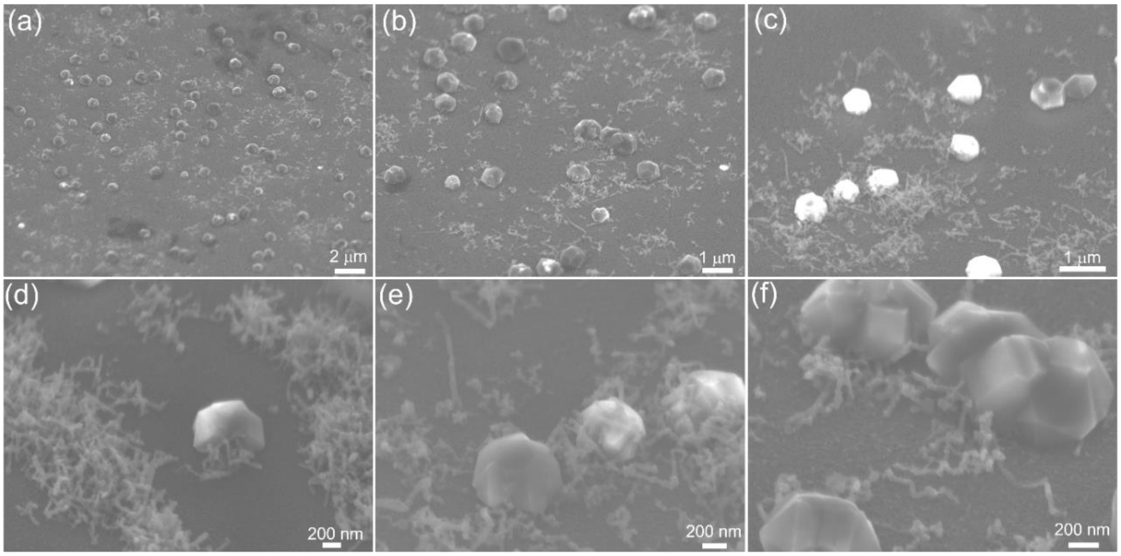

Figure S8. Morphology of the synthesized materials on Si substrate after repeating the growth process for 5 times.

Figure S9 presents the calculated binding energy of C_2, Si-C, and Si_2 molecules. Since the binding energy of Si-C is lower than that of C-C. The C atoms prefer to react with the sputtered Si atoms to form Si-C bond. Hence, the formation of β-SiC is more preferable than graphic C during the early MPCVD process.

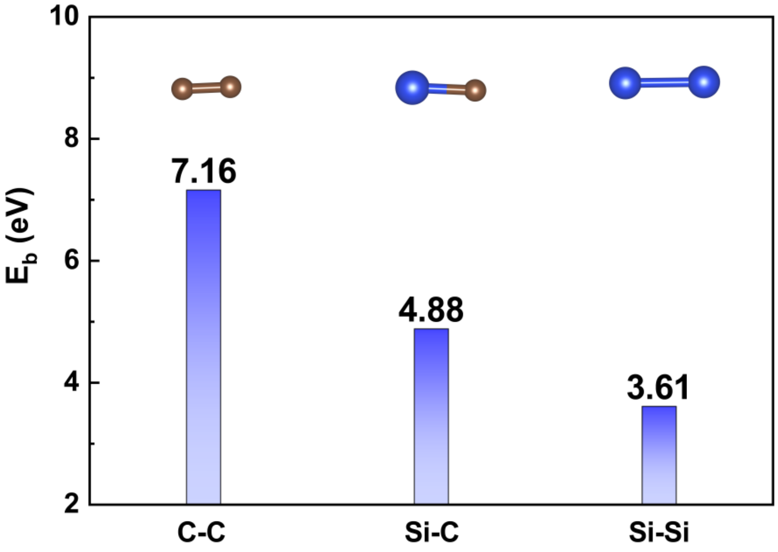

Figure S9. Calculated binding energy of C_2, Si-C and Si_2 molecules.

Influence of β-SiC on the growth of diamond polycrystalline film. Figure S10 presents the interfacial microstructure of the diamond grown on SiO_2/Si(001). The HRTEM image in Figure S10a showed that the β-SiC interlayer is polycrystalline. Figure S10b showed the STEM-EDX mappings of the interfacial structure. Figure S10c presents distinct deposition rate of diamond film on SiO_2/Si(001) and Si(001) substrate.

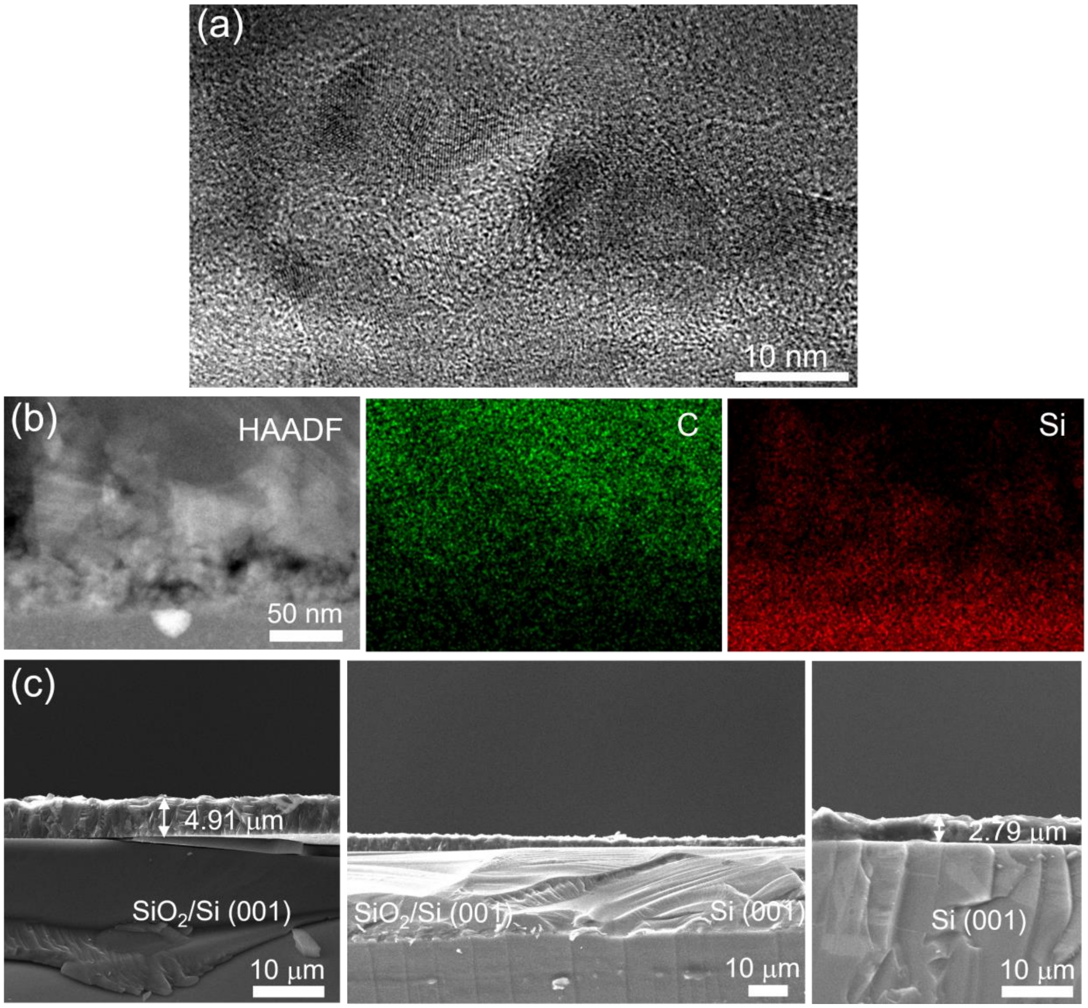

Figure S10. (a) HRTEM image and (b) corresponding EDX mappings of the interfacial structure of diamond film grown on SiO_2/Si (001) substrate. (c) The cross-sectional SEM images presenting the diamond film thickness deposited on SiO_2/Si(001) and Si(001) substrates, respectively.

At the same growth conditions, the obtained diamond film presented discrete (Figure S11b) and continuous (Figure S11d) feature on Si (111) and Si (001) substrate. Since the epitaxial β-SiC nanoislands on Si(111) and Si(001) present distinct morphologies, i.e. with three (Figure S11a) and four (Figure. S11c) exposed {111} surfaces, we infer that it is the morphology of the epitaxial β-SiC nanoislands that affects the subsequent growth of diamond films.

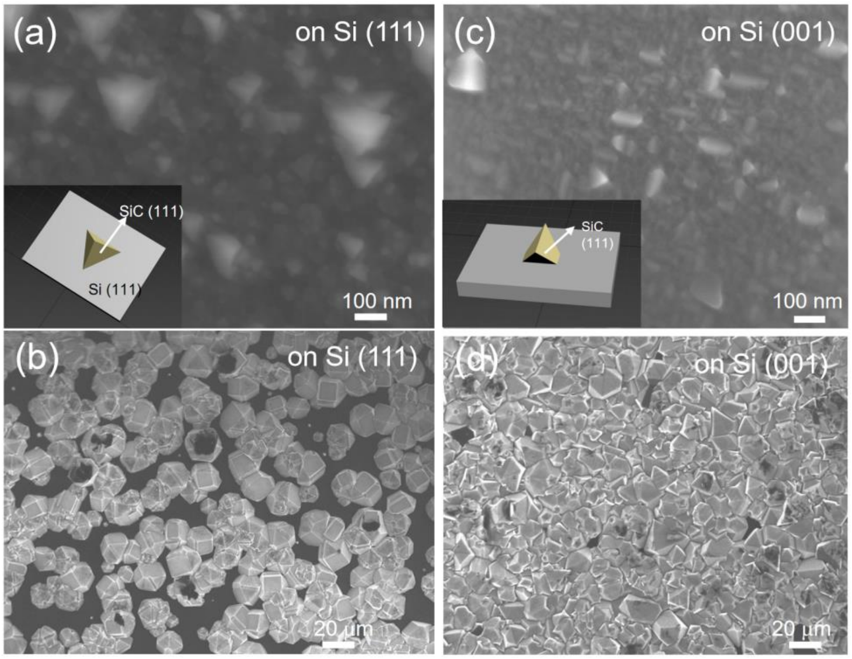

Figure S11. SEM image of the SiC nanocrystals and subsequent synthesized diamond film on Si (111) and Si (001) substrate under the same condition.

Interfacial structural control during MPCVD process. Figure S12 presents the morpholoy and structure of the synthesized diamond film at 3% (Figure S12 a-c), 10% (Figure S12 d-f) and 20% (Figure S12 g-i) CH_4 concentration in CH_4/H_2 mixture.

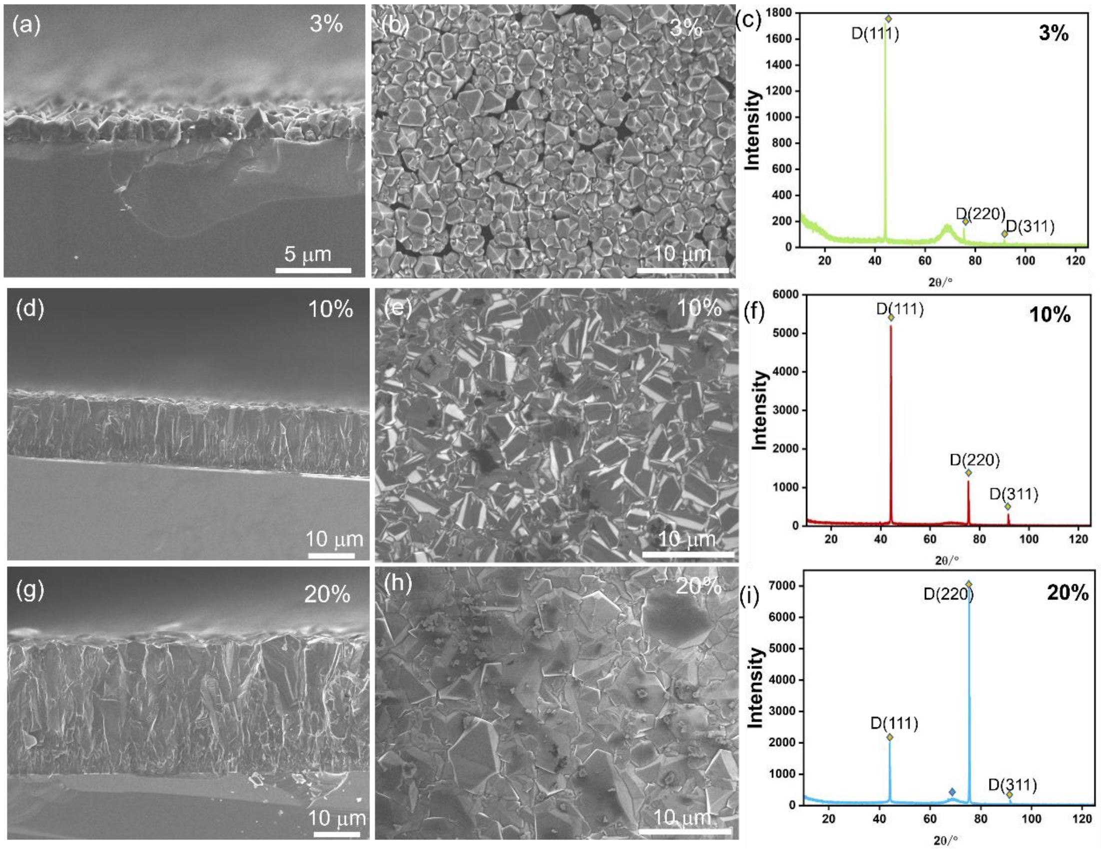

Figure. S12. SEM images and XRD spectra of diamond growth on Si (001) substrate under (a-c) 1%, (d-f) 10% and (g-i) 20% CH_4/H_2 concentration.

To compare the influence of CH_4 concentration on the thickness and orientation of the diamond film grown on Si substrate with different orientations, we synthesized diamond film on Si (001) and Si (111) for under CH_4 concentrations of 10%, 15% and 20%, while keeping other conditions the same. Figure S13 compares the morphology and film thickness of diamond grown on Si (001) and Si (111) substrate. In both cases, the diamond film thickness increases with the CH_4 concentration.

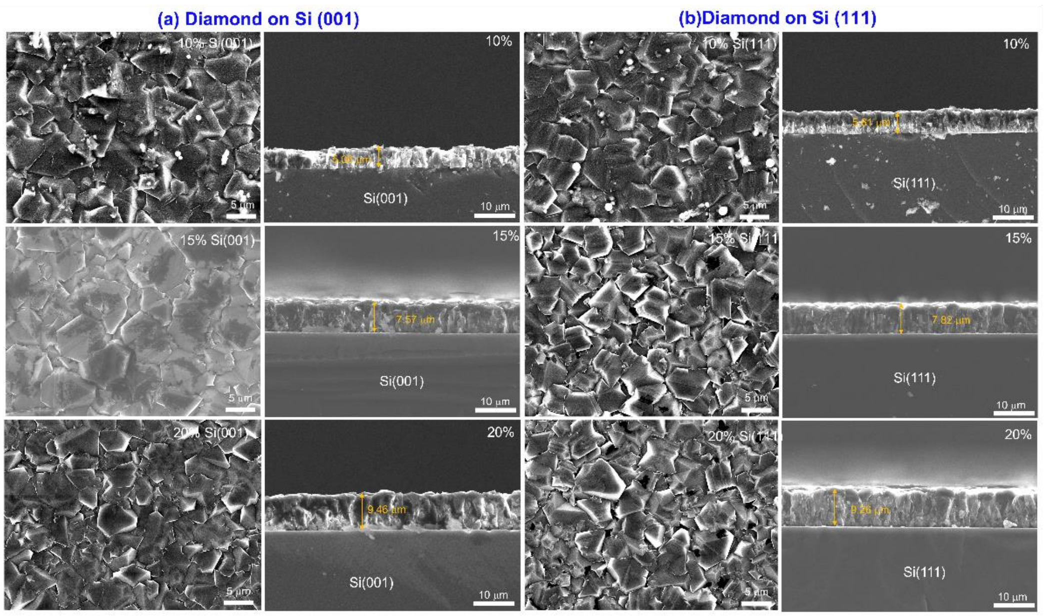

Figure S13 Morphology and thickness characterizations of diamond film grown on (a) Si (001) and (b) Si(111) substrate.

Figure. S14 present the STEM-EDX mappings of the interfacial structure of the diamond film grown at 10% and 20% CH_4/H_2 concentrations.

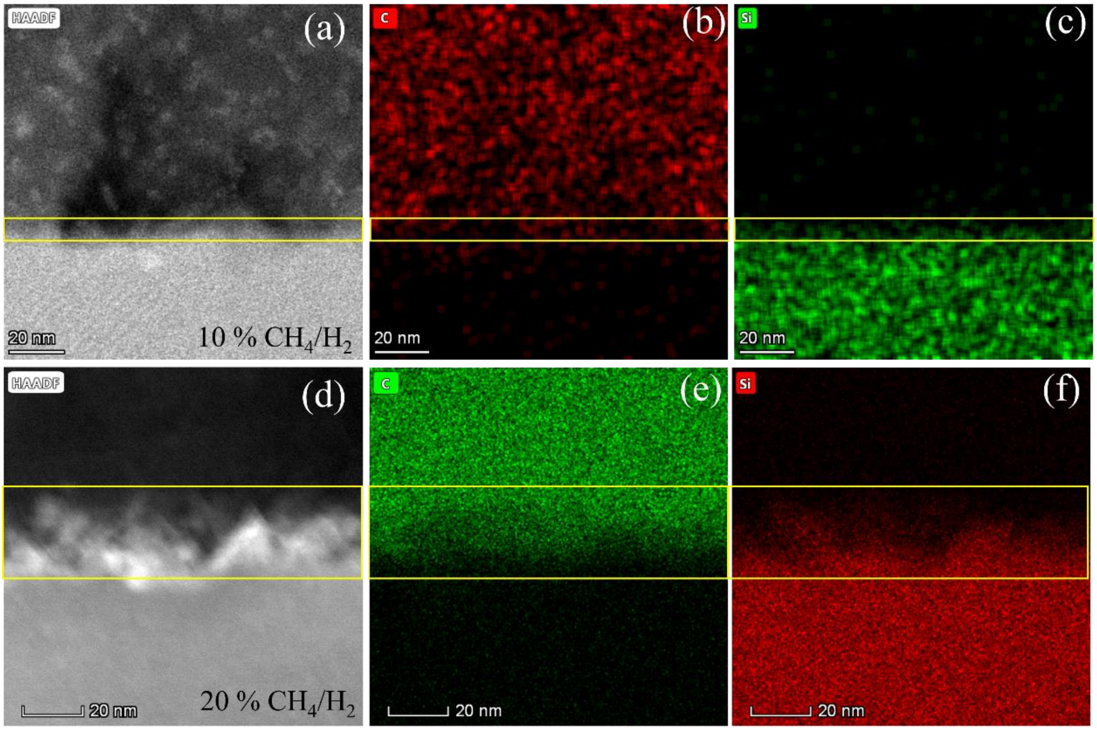

Figure. S14 STEM-EDX mappings of the interfacial structure of diamond film synthesized at 10% and 20% CH_4/H_2 concentrations.

Theoretical calculations showed that the Si atom can hardly react with the C atoms in graphitic carbon to form Si-C bond (Figure. S15).

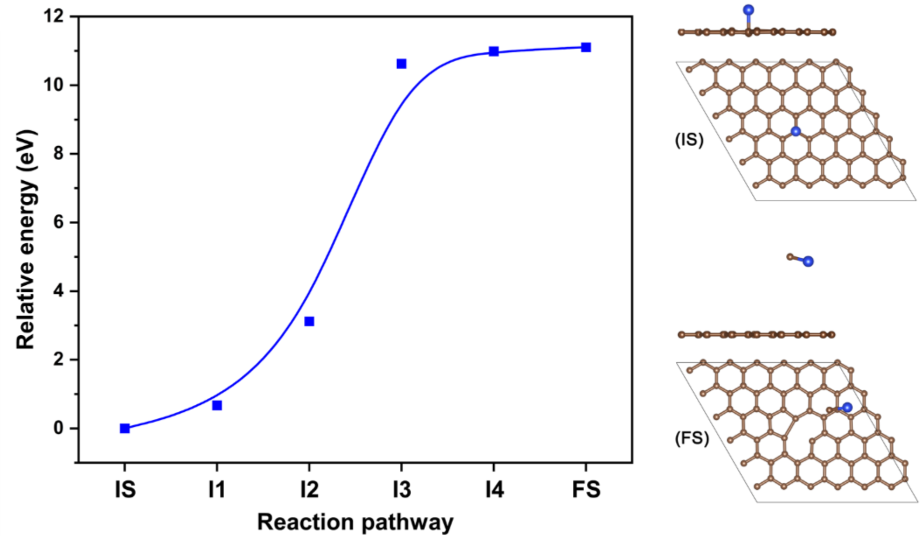

Figure. S15 Minimum energy pathway for Si atom with C atoms in graphene to form Si-C bond.
